# Psychological reactions and insomnia in adults with mental health disorders during the COVID-19 outbreak

**DOI:** 10.1186/s12888-020-03036-7

**Published:** 2021-01-08

**Authors:** Qimeng Sun, Qingsong Qin, Maria Basta, Baixin Chen, Yun Li

**Affiliations:** 1 Department of Sleep Medicine, Shantou University Mental Health Center, Shantou, Guangdong China; 2 Sleep Medicine Center, Shantou University Medical College, Shantou, Guangdong China; 3 Laboratory of Human Virology and Oncology, Shantou University Medical College, Shantou, Guangdong China; 4 Department of Psychiatry, University, Hospital of Heraklion, Crete, Greece

**Keywords:** COVID-19, Stress, anxiety, Depression, Insomnia, Mental health disorders

## Abstract

**Background:**

The 2019 coronavirus disease (COVID-19) has disrupted millions of lives and commerce. We investigated psychological reactions and insomnia during the COVID-19 outbreak in adults with mental health disorders (MDs).

**Methods:**

A self-reported psychological and sleep online survey was conducted in China between February 5th to 19th, 2020. A total of 244 adults with MDs and 1116 controls matched for age, gender and sites were included. Worsened symptoms of anxiety, depressive and insomnia were defined when severity levels shifted to a more severe category compared to pre-COVID-19.

**Results:**

During the COVID-19 outbreak, we found significantly increased prevalence of anxiety (MDs: 54.9% vs. 49.6%, controls: 25.5% vs. 14.3%), depression (MDs: 63.9% vs. 61.5%, controls: 29.9% vs. 21.2%) and insomnia (MDs: 66.0% vs. 57.8%, controls: 31.5% vs. 24.8%) compared to pre-COVID-19 period (all *P*-value < 0.001). Furthermore, adults with MDs had higher odds for developing COVID-19-related stress (OR = 3.41, 95% CI 2.49 ~ 4.67), worsened anxiety (OR = 1.95, 95% CI 1.38 ~ 2.76), depression (OR = 2.04, 95% CI 1.43 ~ 2.93) and insomnia (OR = 2.22, 95% CI 1.53 ~ 3.21) during the COVID-19 outbreak compared to controls. Moreover, higher COVID-19-related stress and lower levels of pre-COVID-19 anxiety, depressive and insomnia symptoms were predictors for worsened anxiety, depression and insomnia in adults with MDs, respectively.

**Conclusions:**

Our findings suggest that adverse psychological reactions and insomnia are more pronounced in adults with mental health disorders during the COVID-19 outbreak, thus more attention need to be provided.

## Background

Since December 2019, an outbreak of a highly contagious, novel coronavirus that causes respiratory illness and severe pneumonia (the 2019 Coronavirus disease, COVID-19) has spread to 200 countries, areas or territories and became a global pandemic. As COVID-19 continued spreading, tens of thousands of lives were lost, and most commercial activities were disrupted [1, 2]. Psychological problems associated with COVID-19, including anxiety, depression, stress, insomnia as well as negative outcomes have drawn a lot of attention from the public as well as medical community [3–7].

Increasing surveys have been conducted all over the world to investigate psychological reactions and sleep changes in the general population [8–10], medical staff [11–13], as well as COVID-19 infected patients [14] and found that prevalence of stress, anxiety, depression and insomnia increased significantly during the COVID-19 pandemic [15–20]. Recently, two systematic reviews and meta-analyses further confirmed the negative impacts of COVID-19 outbreak on psychological reactions and insomnia [21, 22]. Given that patients with mental health disorders (MDs) are more vulnerable to stress-related events [23] and need to cope with their original mental illness, they are less likely to utilize active problem-solving strategies when facing life events [24, 25]. Moreover, recently, we found MDs are a risk factor for developing worsened insomnia during the COVID-19 outbreak in China based on a nationwide survey [16].

Given that people with MDs are often neglected following a disaster, we aimed to investigate the effect of COVID-19 outbreak on stress, anxiety, depression and insomnia in mentally ill patients derived from a national-wide sample in China. In this study, we hypothesized that patients with MDs would be more vulnerable to be affected by the COVID-19 outbreak in terms of psychological reactions and sleep.

## Methods

### Study design

We conducted a nationwide psychological and sleep online survey through the WeChat-based survey program, Questionnaire Star, between February 5th to 14th, 2020. Online questionnaires were sent by the members of the Department of Sleep Medicine, Shantou University Mental Health Center to all people included in their contact lists. All participants need to complete a questionnaire for assessing anxiety, depressive and insomnia symptoms, retrospectively, referring to the time-period prior to the COVID-19 outbreak (January 6th -20th, 2020). Additionally, the same questionnaires were used to assess corresponding symptoms during the COVID-19 outbreak (after January 21st 2020). We chose January 21st,2020 as the cut-off because person-to-person transmission of COVID-19 was confirmed and announced to the public on that date. In addition, nationwide and international lockdown, as well as a series of prevention and control measures have been implemented after that date. The study was approved by the Research Ethics Board of Shantou University Mental Health Center and all methods were carried out in accordance with relevant guidelines and regulations. All participants signed informed consents online and were informed that their personal information was confidential.

### Participants

In this national wide psychological and sleep online survey, a total of 7123 subjects were initially approached. Among those 3772 subjects completed the questionnaires (total response rate: 52.96%). The 3772 subjects were from all 31 provinces in mainland China and Hong Kong Special Administrative (SAR), had mean age of 33.93 ± 10.27 years (range:8–76 years) and 62.4% were females.

In the current study, we used a subgroup of the entire sample of adults (age ≥ 18 years) with a diagnosis of mental health disorder as well as a matched controlled group based on age (± 2 years, range 18–69 years), gender and sites (provinces), to compare psychological reactions and changes of sleep during the COVID-19 outbreak. Mental health disorder was defined by a self-report of prescription of psychiatric medication and/or history of any mental health disorder diagnosis, based on the questionnaire completed by the subjects. No subject with a diagnosis of COVID-19 infection was included in this study. Finally, a total of 244 adults with MDs and 1116 controls were included in the analyses.

### Psychological and sleep assessments

The Impact of Event Scale-Revised (IES-R) questionnaire [26–28] was used to assess subjective stress levels caused by COVID-19, with higher total scores indicating more severe levels of stress. A total score of IES-R ≥ 24 was defined as clinically concerning COVID-19-related stress [26].

Generalized Anxiety Disorder-7 (GAD-7) questionnaire [29, 30] was used to assess anxiety symptoms with higher total scores indicating more severe anxiety. The total score of 5 ~ 9 was defined as mild anxiety, 10 ~ 14 was defined as moderate anxiety, 15 ~ 21 was defined as severe anxiety, and ≤ 4 was defined as no anxiety, respectively. Worsened anxiety symptoms during the COVID-19 outbreak were defined when severity levels shifted to a more severe category compared to pre-COVID-19 levels (i.e., shifted from mild to moderate) based on the clinical cut-off points of the GAD-7. △GAD-7 represents GAD-7 score during outbreak minus pre-COVID-19 GAD-7 score.

Patient Health Questionnaire-9 (PHQ-9) questionnaire [31, 32] was used to assess depressive symptoms with higher total scores indicating more severe depression. The total score of 5 ~ 9 was defined as mild depression, 10 ~ 14 was defined as moderate depression, 15 ~ 19 was defined as moderate to severe depression, 20 ~ 27 was defined as severe depression, and ≤ 4 was defined as no depression, respectively. Worsened depressive symptoms were defined when severity levels during the COVID-19 outbreak shifted to a more severe category compared to pre-COVID-19 levels (i.e., shifted from mild to moderate) based on the clinical cut-off points of the PHQ-9. △PHQ-9 represents PHQ-9 score during outbreak minus pre-COVID-19 PHQ-9 score.

Insomnia Severity Index (ISI) scale [33–35] was used to assess insomnia symptoms with higher total scores indicating more severe insomnia. The total score of 8 ~ 14 was defined as mild insomnia, 15 ~ 21 was defined as moderate insomnia, > 21 was defined as severe insomnia, and ≤ 7 was defined as no insomnia, respectively. Worsened insomnia symptoms were defined when severity levels during the COVID-19 outbreak shifted to a more severe category compared to pre-COVID-19 levels (i.e., shifted from mild to moderate) based on the clinical cut-off points of the ISI. △ISI represents ISI score during outbreak minus pre-COVID-19 ISI score.

Self-reported total sleep time (TST), bedtime and get-up time of pre- and during- the COVID-19 outbreak were also assessed. Time in bed (TIB) was calculated by get-up time minus bedtime. Sleep efficiency (SE) was calculated by (TST/TIB) * 100% [36].

### Sociodemographic and clinical characteristics

Sociodemographic and clinical information including age, gender, site of residence at the time during the COVID-19 outbreak, occupation, marital status, educational levels, and history of MDs were collected based on a standardized questionnaire completed online.

### Statistical analyses

Bivariate comparisons between groups were performed using independent t-test or Mann–Whitney U test for normally and non-normally distributed continuous variables, or using χ^2^ test for categorical variables. Comparisons between pre- and during- COVID-19 outbreak levels of anxiety, depression and sleep were performed using paired t-test or non-parametric Wilcoxon test for normally and non-normally distributed continuous variables. To assess the association between MDs and stress, anxiety, depressive and insomnia symptoms, 4 multivariate adjusted logistic regression models were conducted, respectively. In these regression models, age, gender, occupation, marital status, and educational levels were included as covariates. Furthermore, logistic regression models with age, gender, occupation, marital status, educational levels, pre-COVID-19 total scores of GAD-7, PHQ-9 and ISI and total scores of IES-R during the COVID-19 outbreak as predictors were used to examine risk factors for developing worsened anxiety, depressive and insomnia symptoms during the COVID-19 outbreak in subjects with MDs. Moreover, change in TIB (during-COVID-19 TIB minus pre-COVID-19 TIB) was also added into the regression model as an additional predictor for worsened insomnia symptoms during the COVID-19 outbreak. In order to examine the associations of specific severity of anxiety, depressive and insomnia symptoms prior to the COVID-19 outbreak and corresponding worsened symptoms during the COVID-19 outbreak among adults with MDs, sensitivity analyses were conducted. In the sensitivity analyses, we used logistic regression models with “no-to-mild” anxiety, depressive and insomnia symptoms as predictors and the corresponding worsened symptoms as outcomes while after controlling for potential confounders to examine such associations. SPSS (version 23.0) was used for statistical analysis. A *P* < 0.05 was used to determine statistical significance.

## Results

### Sociodemographic characteristics

Sociodemographic characteristics of the overall sample, as well as adults with and without MDs were shown in Table 1. We included 244 mentally ill patients with a mean age of 33.77 ± 10.87 years and 72.1% females and 1116 controls matched for age, gender, sites and marital status. The included subjects resided in 28 provinces of mainland China and Hong Kong SAR. Among the 244 adults with MDs, 3 (1.2%) subjects had schizophrenia, 13 (5.3%) subjects had bipolar disorder, 86 (35.2%) subjects had depression, 98 (40.2%) subjects had anxiety, 17 (7.0%) subjects had obsessive-compulsive disorder and 27 (11.1%) subjects had other MDs.

**Table 1:** Sociodemographic Characteristics of Study Subjects

	All (*n* = 1360)	Controls (*n* = 1116)	Mental Health Disorders (*n* = 244)	*P*
Age (years)	32.71 ± 9.10	32.48 ± 8.65	33.77 ± 10.87	0.40
Gender (Female, %)	979 (72.0%)	803 (72.0%)	176 (72.1%)	0.96
Occupation				**< 0.001**
Medical staff	347 (25.5%)	287 (25.7%)	60 (24.6%)	
Enterprise and public institution staff	411 (30.2%)	354 (31.7%)	57 (23.4%)	
Teacher	35 (2.6%)	16 (1.4%)	19 (7.8%)	
Student	213 (15.7%)	177 (15.9%)	36 (14.8%)	
Retired or unemployed	102 (7.5%)	84 (7.5%)	18 (7.4%)	
Others	252 (18.5%)	198 (17.7%)	54 (22.1%)	
Marital status (married, %)	810 (59.6%)	668 (59.9%)	142 (58.2%)	0.63
Education levels				**< 0.001**
High school or below	217 (16.0%)	150 (13.4%)	67 (27.5%)	
College	803 (59.0%)	665 (59.6%)	138 (56.6%)	
Master or above	340 (25.0%)	301 (27.0%)	39 (16.0%)	

All values are presented as mean ± SD

Values in bold indicate statistically significant

### Psychological and sleep characteristics

#### Pre-COVID-19 outbreak

Prior to the COVID-19 outbreak, total scores of GAD-7, PHQ-9 and ISI, as well as prevalence of anxiety, depressive and insomnia symptoms were significantly higher in adults with MDs compared to controls (Table 2, all *P*-value< 0.001). Furthermore, adults with MDs reported delayed get up time (*P* = 0.01), prolonged TIB (*P* = 0.006), shorter TST (*P* = 0.002), and reduced SE (*P* < 0.001) compared to controls (Table 2).

**Table 2:** Psychological and Sleep Characteristics of Study Subjects Prior to and During the COVID-19 Outbreak

	Controls			Mental Health Disorders			P^1^	P^2^	P^3^
	Pre-COVID-19	During-COVID-19	△	Pre-COVID-19	During-COVID-19	△			
IES-R		12.03 ± 12.70			21.05 ± 15.93		**< 0.001**		
IES-R ≥ 24 (%)		15.9			38.1			**< 0.001**	
GAD-7	1.75 ± 2.96	2.93 ± 3.90	1.18 ± 2.87	5.51 ± 5.17	6.79 ± 5.96	1.28 ± 4.89	**< 0.001**	**< 0.001**	**< 0.001**
GAD-7 > 4 (%)	14.3	25.5	11.2	49.6	54.9	5.3	**< 0.001**	**< 0.001**	**< 0.001**
PHQ-9	2.36 ± 3.53	3.43 ± 4.38	1.08 ± 3.06	7.25 ± 6.32	8.38 ± 6.70	1.12 ± 4.56	**< 0.001**	**< 0.001**	**< 0.001**
PHQ-9 > 4 (%)	21.1	29.9	8.8	61.5	63.9	2.4	**< 0.001**	**< 0.001**	**< 0.001**
ISI	5.02 ± 4.44	5.90 ± 5.16	0.89 ± 3.30	9.55 ± 6.44	11.00 ± 6.95	1.45 ± 4.76	**< 0.001**	**< 0.001**	**< 0.001**
ISI > 7 (%)	24.8	31.5	6.7	57.8	66.0	8.2	**< 0.001**	**< 0.001**	**< 0.001**
Bed time	23:31 ± 01:02	23:59 ± 01:21	00:28 ± 01:03	23:39 ± 01:28	0:01 ± 01:37	00:22 ± 01:18	0.80	**< 0.001**	**< 0.001**
Get up time	07:29 ± 01:11	08:51 ± 01:45	01:22 ± 01:30	07:51 ± 01:46	08:57 ± 02:14	01:07 ± 01:45	**0.01**	**< 0.001**	**< 0.001**
TIB (h)	7.97 ± 1.20	8.86 ± 1.56	0.89 ± 1.50	8.19 ± 1.46	8.94 ± 1.90	0.74 ± 1.77	**0.006**	**< 0.001**	**< 0.001**
TST (h)	7.20 ± 1.08	7.91 ± 1.59	0.71 ± 1.45	6.93 ± 1.44	7.43 ± 2.07	0.50 ± 1.72	**0.002**	**< 0.001**	**< 0.001**
Sleep efficiency (%)	90.13 ± 10.44	88.73 ± 13.00	−1.39 ± 11.46	84.31 ± 14.44	82.26 ± 16.59	−2.05 ± 13.17	**< 0.001**	**0.005**	**0.006**

All values are presented as mean ± SD; △, during-COVID-19 outbreak values minus pre-COVID-19 outbreak values; *IES-R* The Impact of Event scale-revised, *GAD-7* Generalized Anxiety Disorder-7, *PHQ-9* Patient Health Questionnaire-9, *ISI* Insomnia Severity Index, *TIB* Time in bed, *TST* Total sleep time, *P*^1^, comparisons between Mental health disorder group vs Control group prior to the COVID-19 outbreak; *P*^2^, comparisons within controls before and during the COVID-19 outbreak; *P*^3^, comparisons within patients with mental health disorders before and during the COVID-19 outbreak

Values in bold indicate statistically significant

#### During-COVID-19 vs. pre-COVID-19 outbreak

As shown in Table 2, total scores of GAD-7, PHQ-9 and ISI, prevalence of anxiety, depressive and insomnia symptoms during the acute phase of COVID-19 outbreak increased significantly compared to pre-COVID-19 period in both groups (all *P*-value < 0.001). Furthermore, delayed bed time and get up time, prolonged TIB and TST, and reduced SE were observed during the acute phase of COVID-19 outbreak compared to the pre-COVID-19 period in both groups (all *P*-values < 0.001).

Overall, COVID-19 related stress, worsened symptoms of anxiety, depression and insomnia were more pronounced in adults with MDs compared to controls during the COVID-19 outbreak. As shown in Table 2, the mean values of IES-R (21.05 ± 15.93vs.12.03 ± 12.70, *P*-value < 0.001) and the prevalence of COVID-19-related stress (38.1% vs.15.9%, *P*-value < 0.001) were significantly higher in adults with MDs compared to controls. Furthermore, 24.2% of adults with MDs and 15.1% of controls developed worsened anxiety symptoms based on GAD-7 (*P* = 0.001), 22.1% of adults with MDs and 13.2% of controls developed worsened depressive symptoms based on PHQ-9 (*P* = 0.001), 21.3% of adults with MDs and 11.2% of controls developed worsened insomnia symptoms based on ISI (*P* < 0.001) during the COVID-19 outbreak, respectively.

**Fig. 1:**
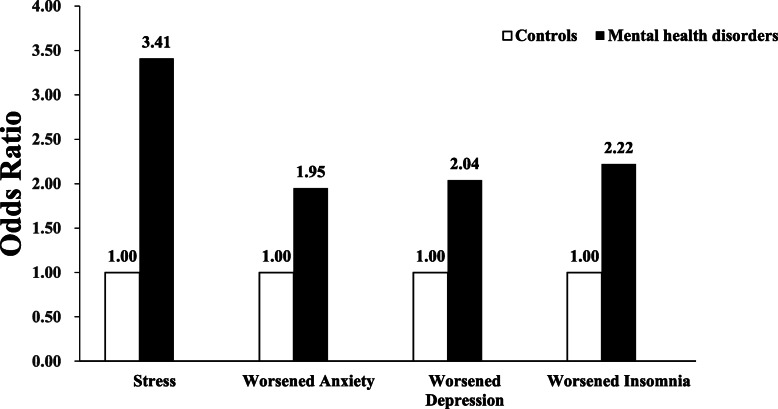
The odds for developing psychological reactions and insomnia in patients with MDs during COVID-19 pandemic. The adjusted odds for developing COVID-19 related stress, worsened anxiety, depressive and insomnia symptoms during the COVID-19 outbreak in patients with mental health disorders compared to controls

Multivariate logistic regression models were used to examine the odds for developing worsened psychological and sleep symptoms in adults with MDs compared to controls during the acute phase of COVID-19 outbreak. After adjusting for age, gender, occupation, marital status, and educational levels, adults with MDs had significantly higher odds for self-reported COVID-19 related stress (OR = 3.41, 95% CI 2.49 ~ 4.67), 1.95-fold (OR = 1.95, 95%CI 1.38–2.76) of odds for developing worsened anxiety symptoms, 2.04-fold (OR = 2.04, 95%CI 1.43–2.93) of odds for developing worsened depressive symptoms and 2.22-fold (OR = 2.22, 95%CI 1.53–3.21) of odds for developing worsened insomnia symptoms during the COVID-19 outbreak compared to controls after adjusting for potential confounders (Fig. 1).

#### Predictors for worsened symptoms in adults with MDs

As shown in Table 3, higher values of IES-R were significantly associated with developed worsened anxiety (OR = 1.13, 95%CI 1.09 ~ 1.17), depressive (OR = 1.11, 95%CI 1.07 ~ 1.14), and insomnia symptoms (OR = 1.05, 95%CI 1.03 ~ 1.08) during the COVID-19 outbreak in adults with MDs. Furthermore, lower levels of anxiety (OR = 0.86, 95%CI 0.75 ~ 0.99) and depressive (OR = 0.90, 95%CI 0.80 ~ 0.96) symptoms prior to COVID-19 outbreak predicted worsened anxiety and depressive symptoms during the COVID-19 outbreak, respectively. Moreover, worsened insomnia symptoms were significantly associated with prolonged TIB (OR = 1.23, 95%CI 1.02 ~ 1.49) during the outbreak and lower levels of insomnia symptoms prior to the COVID-19 outbreak (OR = 0.91, 95%CI 0.84 ~ 0.98). No significant association between sociodemographic characteristics and worsened anxiety, depressive and insomnia symptoms was observed in adults with MDs.

**Table 3:** Risk factors for Worsened Emotion and Insomnia in Adults with MDs during the COVID-19 Outbreak

Variable	Worsened Anxiety		Worsened Depression		Worsened Insomnia	
	OR	95%CI	OR	95%CI	OR	95%CI
**Age**	0.98	0.93 ~ 1.02	0.97	0.92 ~ 1.01	0.99	0.95 ~ 1.03
**Gender**						
Female	Reference					
Male	0.90	0.38 ~ 2.13	1.30	0.56 ~ 3.02	0.72	0.32 ~ 1.61
**Occupation**	0.92	0.73 ~ 1.16	0.87	0.69 ~ 1.10	1.03	0.84 ~ 1.28
**Married**						
Yes	Reference					
No	0.86	0.35 ~ 2.09	0.77	0.31 ~ 1.94	0.61	0.26 ~ 1.42
**Education levels**						
High school and below	Reference					
College	0.86	0.32 ~ 2.30	1.11	0.42 ~ 2.94	2.27	0.90 ~ 5.74
Master and above	1.23	0.37 ~ 4.10	0.64	0.16 ~ 2.47	1.25	0.34 ~ 4.61
**IES-R**	**1.13**	**1.09 ~ 1.17**	**1.11**	**1.07 ~ 1.14**	**1.05**	**1.03 ~ 1.08**
**GAD-7 prior to outbreak**	**0.86**	**0.75 ~ 0.99**	0.96	0.84 ~ 1.10	0.99	0.88 ~ 1.12
**PHQ-9 prior to outbreak**	0.95	0.85 ~ 1.06	**0.90**	**0.80 ~ 0.96**	1.02	0.93 ~ 1.13
**ISI prior to outbreak**	0.98	0.90 ~ 1.07	1.01	0.93 ~ 1.10	**0.91**	**0.84 ~ 0.98**
**△TIB**	**–**	**–**	**–**	**–**	**1.23**	**1.02 ~ 1.49**

*IES-R* The Impact of Event scale-revised, *GAD-7* Generalized Anxiety Disorder-7, *PHQ-9* Patient Health Questionnaire-9, *ISI* Insomnia Severity Index, *△TIB* Total in-bed time during the outbreak minus pre-COVID-19 total in-bed time

Values in bold indicate statistically significant

In sensitivity analyses, findings of logistic regression models suggested that among adults with MDs, adults with no to mild anxiety symptoms prior to the COVID-19 outbreak (OR = 6.02, 95% CI 1.47 ~ 24.67) were at risk for worsened anxiety symptoms during the COVID-19 outbreak after controlling for age, gender, occupation, marital status, education levels, IES-R scores, baseline PHQ-9 and ISI scores. Regarding depressive symptoms, we found adults with MDs with no to mild depressive symptoms prior to the COVID-19 outbreak (OR = 15.73, 95% CI 3.85 ~ 64.29) were at risk for worsened depressive symptoms during the COVID-19 outbreak after controlling for age, gender, occupation, marital status, education levels, IES-R scores, baseline GAD-7 and ISI scores. Regarding insomnia symptoms, we found adults with MDs with no to mild insomnia symptoms prior to the COVID-19 outbreak (OR = 10.16, 95% CI 2.59 ~ 39.85) were at risk for worsened insomnia symptoms during the COVID-19 outbreak after controlling for age, gender, occupation, marital status, education levels, IES-R scores, baseline GAD-7 and PHQ-9 scores.

## Discussion

The findings of this study suggest that stress, severity of anxiety, depressive and insomnia symptoms increased significantly during the COVID-19 outbreak, and these findings are more pronounced in adults with MDs compared to controls. Among adults with MDs, deterioration of psychological and insomnia symptoms during the COVID-19 outbreak were associated with COVID-19 outbreak related stress. Furthermore, lower levels of pre-COVID-19 anxiety, depressive and insomnia symptoms predicted higher deterioration of these symptoms during the COVID-19 outbreak.

The anxiety, depressive and insomnia levels in the mental health disorder group were significantly higher than those in the control group. People with MDs had significant higher values of IES-R compared to controls, which is consistent with a recent study on psychiatric patients [28], suggesting that people with MDs are more likely to develop PTSD after the COVID-19 outbreak, and more attention regarding follow-up interview and timely intervention need to be provided to these population.

In this study, as expected, significantly elevated stress, depressive and insomnia symptoms were observed during the acute phase of COVID-19 outbreak in adults with and without MDs. Consistent with previous studies, reporting that 16 ~ 28% of the subjects had anxiety or depression, and about 8% of the subjects had obvious COVID-19 related stress during the COVID-19 outbreak [18]. These may be associated with the nationwide COVID-19 pandemic itself, fear of getting infected and the rapidly increasing number of cases, delayed work and school, economic related stress, travel restrictions and changes in daily life [37].

We also found that adults with MDs were more likely to present deterioration of psychological symptoms during the COVID-19 outbreak period. Specifically, adults with MDs had higher odds for developing COVID-19 stress (OR = 3.41), worsened anxiety (OR = 1.95), depressive (OR = 2.04) and insomnia (OR = 2.22) symptoms during the COVID-19 outbreak compared to controls. It has been reported that patients with MDs are more vulnerable to be influenced by stressful events [23–25]. Recently, Hao et al. reported that psychiatric patients presented more severe stress, anxiety, depressive and insomnia symptoms compared to healthy controls during the COVID-19 outbreak [28]. The reasons for worsened psychological reactions and insomnia in patients with MDs are not well established. They may be associated with increased vulnerability to stress among patients with MDs, resulting in relapses or deterioration of an already existing mental health issue. In addition, many people with MDs attend regular outpatient visits for evaluations and medications’ prescriptions. However, nationwide regulations on travel and quarantine resulted in suspended hospital visits. Based on a psychiatric outpatient population-based survey during the COVID-19 outbreak, Zhou et al. reported that 22.0% of patients diagnosed with mental disorders could not receive routine psychiatric care because of suspended hospital visits [38]. Cessation or irregular administration of medications could be another major reason for emotional status and insomnia deterioration in mentally ill patients.

Another finding of our study was that anxiety, depressive and insomnia symptoms deterioration among adults with MDs were positively related with COVID-related stress. Previous studies showed that stress had adverse effects on the onset of MDs, and patients with MDs tended to utilize more maladaptive coping strategies when faced stress [23, 39]. In our study, 197 out of 244 (80.7%) patients with MDs were diagnosed with affective/anxiety disorders, while only 3 out of them (1.2%) were diagnosed with schizophrenia. Patients with affective/anxiety disorders are more concerned about external environment than patients with negative symptoms of schizophrenia [40, 41]. This may suggest that emotional and insomnia aggravation in patients with affective/anxiety disorders is mainly due to the COVID-19-related issues and drugs, while withdrawal or irregular medication may be the main cause of symptoms exacerbation or relapse in patients with schizophrenia and other MDs.

Interestingly, no to mild levels of anxiety, depressive and insomnia symptoms at the pre-COVID-19 period in adults with MDs were associated with increased risk for worsened anxiety, depressive and insomnia symptoms during COVID-19 outbreak. These findings may be explained by the ceiling effect, i.e., the higher the initial level of psychopathology, the less the worsening may become. Besides, these findings may also be associated with the deterioration or recurrence of MDs due to not receiving routine psychiatric care caused by cancelation of hospital visits in patients in stable condition [38]. Moreover, prolonged TIB during the COVID-19 was significantly associated with worsened insomnia symptoms, which suggested that poor sleep hygiene was a risk factor for insomnia during the COVID-19 outbreak.

In our study demographic factors such as female gender or older age are not identified as significant predictors of more pronounced worsening of psychological symptoms in adults with MDs. Unlike our study, previous studies reported that females and older subjects manifest worse psychological reactions during the COVID-19 outbreak [15, 17]. Lack of such associations in our study may be explained by the fact that our sample consisted predominantly by women (72.0%) and middle-aged people (only 1.0% subjects ≥60 years).

### Clinical implications

Our findings highlight the importance of online and/or hotline psychological intervention services such as cognitive behavior therapy and mindfulness based therapy provided not only to the general population but also to mental health patients that seem to be more vulnerable to exacerbation of their symptoms [42]. This group of patients is often neglected following a disaster, and more attention, including timely psychological interventions and telemedicine need to be provided as soon as possible. Furthermore, since psychological problems may persist after the outbreak period, long term psychological follow-up and intervention service should be considered. COVID-19 is the third pandemic caused by a coronavirus [43]. By far, it has caused wider infection and much more fatalities than its two predecessors (Severe Acute Respiratory Syndrome Coronavirus and Middle East Respiratory Syndrome Coronavirus). Scientists believe that this kind of pandemic can happen again based on the biological features of coronaviruses. Establishing a nationwide, online psychological [44, 45], stress and sleep management and intervention system to prevent and mitigate the immediate and long-term effects of significant traumatic events on psychological health and sleep seems to be of great importance.

### Strengths and limitations

There are several strengths of this study. Firstly, anxiety, depressive and insomnia symptoms were assessed before and during the COVID-19 outbreak. Additionally, validated, broadly used scales were used to examine depressive, and anxiety symptoms as well as subjective sleep. Furthermore, the survey covered most regions of China with a relatively large sample size. However, several limitations should be acknowledged and taken into account when interpreting our results. First, because the study population was not restricted in this research and it is an open, voluntary online survey through acquaintance transmission, the response rate is only 52.96% with no reasons provided by subjects without feedbacks. However, this is within the response rate range of other online surveys [15, 38]. Second, most subjects in our research were young–middle aged (only 1.0% subjects ≥60 years) since the applications used for recruitment were more accessible to younger people. As a result, our findings may not reflect older ages and the age-effect cannot be examined. Third, since most patients in our research had affective/anxiety disorders (80.7%), the corresponding results may not be representative of other MDs such as psychoses.

## Conclusions

In summary, stress, anxiety, depressive and insomnia symptoms significantly deteriorated during the COVID-19 outbreak and findings were more profound in adults with MDs. Our data demonstrate that in addition to efforts focused on the prevention and treatment of COVID-19, the widespread psychological and insomnia problems also need to be addressed through psychological interventions such as cognitive behavior therapy and mindfulness-based therapy, especially in people with MDs, to alleviate their related symptoms and prevent relapse.

## Data Availability

The datasets that were generated analyzed for the current study are not publicly available as the author does not have permission to share the data.

## Abbreviations

CI: Confidence Interval
COVID-19: The 2019 Coronavirus Disease
GAD-7: Generalized Anxiety Disorder-7
IES-R: The Impact of Event Scale-Revise
ISI: Insomnia Severity Index
MDs: Mental Health Disorders
OR: Odds Ratio
PHQ-9: Patient Health Questionnaire-9
PTSD: post-traumatic stress disorder
SAR: Special Administrative
TIB: Time in Bed
SE: Sleep Efficiency
TST: Total Sleep Time
